# Efficacy and safety of acupoint application in the treatment of ulcerative colitis: A systematic review and meta-analysis

**DOI:** 10.1097/MD.0000000000034489

**Published:** 2023-08-18

**Authors:** Yaling Tong, Yunfeng Yu, Shuang Yin, Shanzhi Lin, Yun Chen, Xuan Su

**Affiliations:** a Nanhai District People’s Hospital of Foshan, Foshan, China; b College of Chinese Medicine, Hunan University of Chinese Medicine, Changsha, China; c Guangdong Integrated Traditional Chinese and Western Medicine Hospital affiliated to Guangzhou University of Chinese Medicine, Guangzhou, China; d The Ninth People’s Hospital of Nanhai District, Foshan, China.

**Keywords:** acupoint application, harbord, meta-analysis, trial sequential analysis, ulcerative colitis

## Abstract

**Background:**

The efficacy of acupoint application in the treatment of ulcerative colitis (UC) is still controversial. The purpose of this study is to systematically evaluate the clinical efficacy and safety of acupoint application in the treatment of ulcerative colitis.

**Methods:**

The databases of China National Knowledge Infrastructure (CNKI), Chinese Biology Medicine (CBM), VIP, Wanfang, Embase, PubMed, the Cochrane Library and Web of Science were searched. The time limit was from the establishment of the database to July 2022. The published randomized controlled trials of acupoint application in the treatment of UC were analyzed by meta-analysis and trial sequential analysis.

**Results:**

A total of 13 studies were included, with a total sample size of 878 cases. Compared with conventional western medicine, acupoint application can effectively improve the effective rates of clinical comprehensive (risk ratio [RR] 1.13, 95% confidence interval [CI] 1.06–1.20, *P* = .0003), syndrome (RR 1.13, 95% CI 1.03–1.24, *P* = .009), and interleukin-4 (IL-4) (mean differences 2.62, 95% CI 1.96–3.28, *P* < .00001) in the treatment of UC, and reduce interferon-γ (mean differences *−*5.38, 95% CI *−*6.81 to *−*3.94, *P* < .00001). The effective rates of colonoscopy (RR 0.94, 95% CI 0.84–1.05, *P* = .25), pathological examination (RR 1.04, 95% CI 0.90–1.20, *P* = .60) and rate of adverse reaction (RR 0.55, 95% CI 0.25–1.21, *P* = .14) were the same. Trial sequential analysis indicated that the benefits of effective rates of clinical comprehensive and syndrome, IL-4, and interferon-γ were conclusive. Harbord regression showed no publication bias (*P* = .98). The evaluation of evidence quality suggested that the evidence quality of effective rates of clinical comprehensive and syndrome was moderate and the evidence quality of other indicators was low or very low.

**Conclusion:**

Acupoint application is a safe and effective method for the treatment of UC, and has the prospect of clinical application.

## 1. Introduction

Ulcerative colitis (UC) is a chronic nonspecific inflammatory disease confined to the mucosa and submucosa of the colon and rectum, which is formed by a combination of genetic background and environmental factors.^[1]^ UC has become a common and frequently-occurring disease worldwide.^[2]^ It is estimated that the incidence of UC in the Chinese population is about 11.6/100,000,^[3]^ and it shows an increasing trend year by year.^[4]^ The typical symptoms of UC are abdominal pain, tenesmus, mucous pus and bloody stool,^[5]^ and even systemic symptoms or involvement of extraintestinal organs,^[6,7]^ which seriously endangers human health. Although anti-inflammatory and immuneomodulatory measures have delayed the progression of UC to a certain extent, adverse drug reactions such as nausea, vomiting, headache, and anemia and recurrence after recovery are still difficult problems for clinicians.^[8,9]^ The intervention of novel therapeutic strategies is an urgent need to. It has been reported that acupoint application is called upon to become a new strategy for the treatment of UC due to its significantly better clinical efficacy than conventional western medicine.^[10]^ However, there are also studies showing that the effective rate of clinical comprehensive of acupoint application in the treatment of UC is equivalent to that of sulfasalazine.^[11]^ The clinical efficacy of acupoint application in the treatment of UC is still controversial. Thus, this study took acupoint application as the object, and used the methods of meta-analysis and trial sequential analysis (TSA) to evaluate the clinical efficacy and safety of acupoint application in the treatment of UC, so as to provide evidence-based basis for the clinical application of acupoint application.

## 2. Materials and methods

### 2.1. Search strategies

In the databases of China National Knowledge Infrastructure (CNKI, https://www.cnki.net/), Chinese Biology Medicine (CBM, http://www.sinomed.ac.cn/index.jsp), VIP (http://www.cqvip.com/), WanFang (https://www.wanfangdata.com.cn/), Embase (https://www.embase.com/), PubMed (https://pubmed.ncbi.nlm.nih.gov/), the Cochrane Library (https://www.cochranelibrary.com/), and Web of Science (https://www.webofscience.com/), we searched for clinical studies on acupoint application in the treatment of ulcerative colitis. The time frame for these studies is limited from the start of the database to July 2022. English subject terms included acupoint application and ulcerative colitis, and Chinese subject terms included xuewei tiefu, kuiyangxing jiechangyan (the Chinese name of ulcerative colitis). On the basis of subject headings, Chinese free words are expanded with CNKI and CBM, and English free words are expanded with MeSH database, and then subject headings and free words are combined for retrieval. This is a systematic review and meta-analysis. All data are derived from published literature and do not involve ethical issues.

### 2.2. Inclusion and exclusion criteria

Inclusion criteria:

1. Type of Subjects: Randomised controlled trial.
2. Participants: Consistent with the basic diagnosis of UC.^[1]^
3. Interventions: The patients in the experimental group were given acupoint application, while the patients in the control group were given conventional western medicines (sulfasalazine, mesalazine), and the two groups were given the same course of treatment.
4. Outcomes: The effective rate of clinical comprehensive was used as the primary efficacy endpoint. The effective rates of syndrome, colonoscopy and pathological examination, interleukin-4 (IL-4) and interferon-γ (IFN-γ) were the secondary efficacy endpoints. And the rate of adverse reaction was the safety endpoint. The effective rate of clinical comprehensive referred to the percentage of patients who improve in symptoms, endoscopic presentation and pathology. The effective rate of syndrome referred to the percentage of patients who improve in symptoms.

Exclusion criteria:

1. Reviews, animal experiments, case reports, etc.
2. Repeated published research results.
3. Research results published in abstract form.
4. The subjects included in the study covered special populations such as pregnant women or those with other serious diseases.

### 2.3. Study screening, data statistics and quality evaluation

Firstly, the basic literature retrieved from each database was imported into Endnote X9, and the literature was finally determined after removing duplicates, checking the title and abstract, and reviewing the full text according to the inclusion criteria. Secondly, the included literature was classified and sorted, and the basic characteristics such as author, year, sample size, intervention measures, treatment course, and outcome indicators were extracted and entered into the data statistics table. Thirdly, risk of bias was assessed against the required items using the Cochrane Risk of bias tool. All work was carried out independently by 2 investigators, and any objections were adjudicated by a third investigator.

### 2.4. Statistical analysis

Meta-analysis was performed using Revman5.3, and the risk ratio (RR) and 95% confidence interval (95% CI) were used as the effect size for dichotomous variables. For continuous variables, mean differences (MD) and 95% CI were used as effect sizes. Heterogeneity analysis was based on *I*^2^-test and *Q*-test. If *I*^2^ < 50% and *P* > .1, the heterogeneity was small, and the fixed-effects model was used for analysis; otherwise, the random-effects model was used for analysis. If significant heterogeneity existed, subgroup and sensitivity analyses were used to find sources of heterogeneity. The results were robust if subgroup analysis showed no significant heterogeneity within each subgroup or sensitivity analysis showed no significant change in the combined results after study-by-study exclusion. TSA0.9.5.10 Beta software was used for TSA. If the cumulative *Z* value exceeds the required information size or the TSA threshold, the original result is conclusive. Publication bias was assessed by Stata15.0 software. If Harbord regression showed *P* > .1, there was no publication bias. GRADEpro3.6 software was used to evaluate the quality of evidence, and the evaluation method was based on the GRADE Evidence Evaluation Guidelines.

## 3. Results

### 3.1. Literature search results

**Figure 1.**
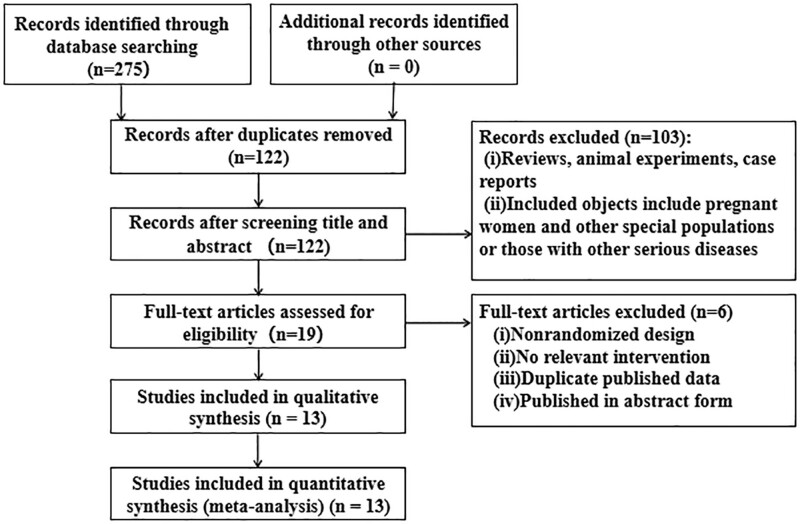
The flow chart of literature screening.

A total of 275 papers were obtained, and 13 papers were included after eliminating duplicates and screening^[12–24]^ (Fig. 1). The included studies were all in Chinese, covering 13 clinical studies with a total sample size of 878 cases, including 440 cases in the experimental group and 438 cases in the control group.

### 3.2. Basic characteristics of included studies

A total of 13 clinical studies were included,^[12–24]^ published between 2002 and 2015, all of which were published in full text. The research centers were all in China, and the average age of the included subjects was 39.2 years old. The average male ratio was 52.3%, and the average disease duration was 4.7 years. The conventional western medicine in the control group was sulfasalazine. The basic characteristics are shown in Table 1.

**Table 1:** Basic characteristics of the included studies.

Author and year	Group	Usage and dosage	Sample size	Male/female	Age/year	Course of disease/year	Severity of illness	Course of treatment/week	Outcomes
FENG G 2002	Acupoint application	qd	30	17/13	37.35	3.35	/	4	①
	Sulfasalazine	1st week: increase from 0.5g each time by 0.25 g to 2.0 g bid; 2nd week: 2.0 g tid; 3rd week: decrease from 2.0 g each time by 0.2 5g to 1.0 g tid	30	14/16	38.35	3.87	/		
YI X 2006	Acupoint application	qd	30	19/11	48.41	3.62	Low: 16 Moderate: 11 Severe:3	4	①⑦
	Sulfasalazine	1st week: increase from 0.5 g each time by 0.25 g to 2.0 g bid; 2nd week: 2.0 g tid; 3rd week:decrease from 2.0 g each time by 0.25 g to 1.0 g tid	28	15/13	46.15	4.15	Low: 14 Moderate: 12 Severe:2		
YU Y 2011	Acupoint application	q5d	30	20/10	38.72	4.6	Low: 22 Moderate: 8	8	①③④⑤⑦
	Sulfasalazine	4.0 g/d tid	30	17/13	36.78	5.26	Low: 24 Moderate: 6		
CHEN C 2012	Acupoint application	q5d	30	20/10	38.7	4.6	/	8	①⑦
	Sulfasalazine	4.0 g/d tid	30	17/13	36.8	5.3	/		
TIAN J 2012	Acupoint application	qd	53	27/26	/	/	Low: 18 Moderate: 35	8	①
	Sulfasalazine	0.75 g tid	53	19/34	/	/	/		
ZHONG X 2012	Acupoint application	q2d	35	20/15	37.8	/	/	8	①
	Sulfasalazine	3.0–-4.0 g/dtid/qid	35	17/18	36.6	/	/		
GUO W 2013	Acupoint application	q5d	20	14/6	40.85	3.35	Low: 12 Moderate:8	8	①④⑤⑥⑦
	Sulfasalazine	4.0–6.0 g/d qid	20	12/8	42.9	3.7	Low: 13 Moderate:7		
HE R 2013	Acupoint application	twice a week	60	26/34	45.27	/	/	48	①
	Sulfasalazine	0.5 g qid	60	20/40	43.2	/	/		
HUANG L 2013	Acupoint application	q2d	20	11/9	33	6.6	Low: 14 Moderate:6	8	①⑤⑥⑦
	Sulfasalazine	1.0 g qid	20	12/8	32	7.5	Low: 13 Moderate:7		
ZOU G 2013	Acupoint application	qd	30	18/12	34.72	5.3	/	4	①⑦
	Sulfasalazine	1st week: increase from 0.5 g each time by 0.25 g to 2.0 g bid; 2nd week: 2.0 g tid; 3rd week: decrease from 2.0 g each time by 0.25 g to 1.0 g tid	30	17/13	35.78	5.26	/		
ZOU J 2013	Acupoint application	q5d	50	23/27	37.92	4.7	Low:36 Moderate:14	8	①②③④⑤⑥⑦
	Sulfasalazine	4.0 g/d tid	50	24/26	37.78	4.5	Low: 34 Moderate:16		
CAI C 2014	Acupoint application	q5d	22	/	/	/	/	4	①
	Sulfasalazine	4.0 g/d tid	22	/	/	/	/		
CAI C 2015	Acupoint application	q5d	30	/	/	/	/	8	①③
	Sulfasalazine	1.0 g qid	30	/	/	/	/		

① represents the clinical symptom cure rate; ② represents the endoscopic cure rate; ③ represents the total effective rate of clinical symptoms; ④ represents the endoscopic total effective rate; ⑤ represents the adverse event rate.

### 3.3. Risk of bias assessment

**Figure 2.**
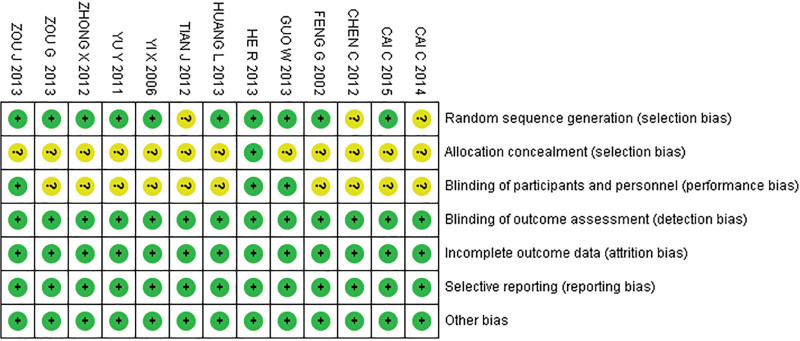
Risk of bias assessment diagram.

Risk of bias in included studies was assessed using the Cochrane Bias Assessment Tool. Results showed randomization methods in 3 studies, allocation concealment in 12 studies, unclear risk of intervention blinding of participants in 10 studies, and low risk of bias in the remaining domains (Fig. 2).

### 3.4. Meta-analysis results

#### 3.4.1. Primary efficacy endpoint.

**Figure 3.**
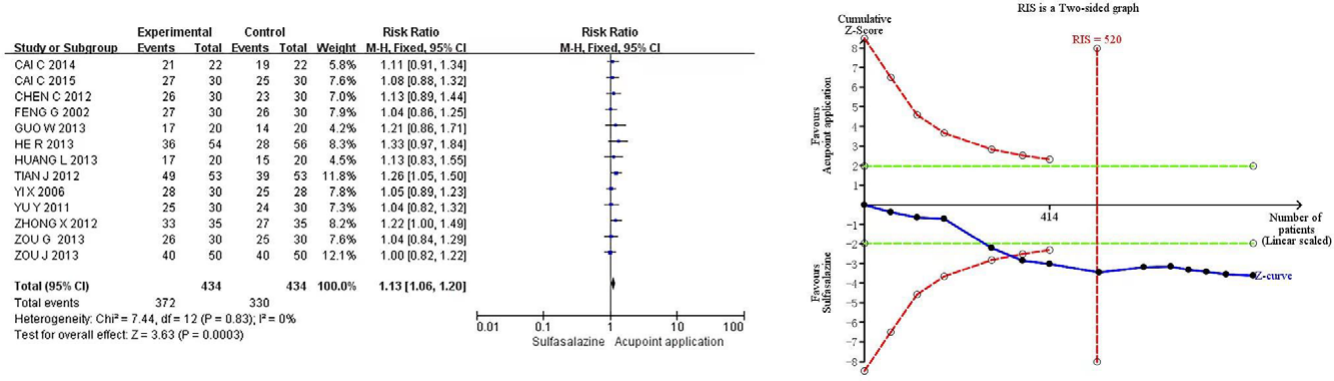
Meta-analysis and TSA of effective rate of clinical comprehensive of acupoint application in the treatment of UC. 95% CI = 95% confidence interval, TSA = trial sequential analysis, UC = ulcerative colitis.

Thirteen studies^[12–24]^ reported the primary efficacy endpoint (effective rate of clinical comprehensive). The *Q*-test and *I*^2^-test indicated that the heterogeneity was small (*P* = .83, *I*^2^ = 0%), so the fixed effect model was used for analysis. The results showed that, compared with sulfasalazine, acupoint application could effectively improve the effective rate of clinical comprehensive in the treatment of UC (RR 1.13, 95% CI 1.06–1.20, *P* = .0003). TSA showed that the cumulative Z-score for the effective rate of clinical comprehensive crossed the TSA threshold in the fifth study, suggesting that the current results were conclusive (Fig. 3).

#### 3.4.2. Secondary efficacy endpoint.

Secondary efficacy endpoints include effective rates of syndrome,^[13,14,20–22,24]^ colonoscopy,^[14,18,22,24]^ pathological examination,^[14,18,22]^ IL- 4,^[14,18,20,22]^ IFN-γ.^[15,18,20,22]^ The *Q*-test and *I*^2^-test showed that the heterogeneities of the 5 indicators were small, and the fixed effect models were used for analysis. The results showed that, compared with sulfasalazine, acupoint application could significantly improve the effective rate of syndrome (RR 1.13, 95% CI 1.03–1.24, *P* = .009) and IL-4 (MD 2.62, 95% CI 1.96–3.28, *P* < .00001) in the treatment of UC, and significantly reduce IFN-γ (MD *−*5.38, 95% CI *−*6.81 to *−*3.94, *P* < .00001). The effective rates of colonoscopy (RR 0.94, 95% CI 0.84–1.05, *P* = .25) and pathological examination (RR 1.04, 95% CI 0.90–1.20, *P* = .60) were comparable. TSA demonstrated that the benefits of effective rate of syndrome, IL-4, and IFN-γ were conclusive, while the results of effective rate of colonoscopy and pathological examination were inconclusive and need to be confirmed by more studies. As are shown in Table 2.

**Table 2:** Meta-analysis and TSA of secondary efficacy endpoints of acupoint application in the treatment of UC.

Outcomes	*I*^2^/%	Analytical model	RR/MD (95% CI)	*P*	TSA
Effective rate of syndrome	0	FEM	1.13 (1.03–1.24)	.009	Yes
Colonoscopy effective rate	27	FEM	0.94 (0.84–1.05)	.25	No
Pathological examination effective rate	0	FEM	1.04 (0.90–1.20)	.60	No
IL-4	0	FEM	2.62 (1.96–3.28)	<.00001	Yes
IFN-γ	40	FEM	−5.38 (−6.81 to −3.94)	<.00001	Yes

FEM = fixed-effects model, IFN-γ = interferon-γ, IL-4 = interleukin-4, MD = mean differences, RR = risk ratio, TSA = trial sequential analysis, UC = ulcerative colitis.

#### 3.4.3. Rate of adverse reaction.

**Figure 4.**
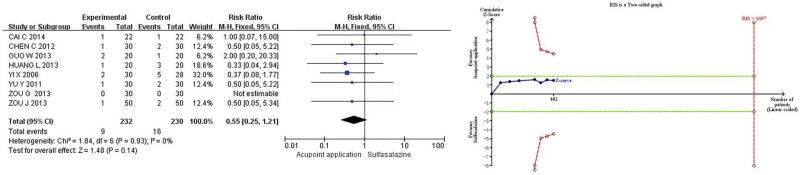
Meta-analysis and TSA of the rate of adverse reaction of acupoint application in the treatment of UC. 95% CI = 95% confidence interval, TSA = trial sequential analysis, UC = ulcerative colitis.

Eight studies^[13–15,18,20–23]^ reported safety endpoints (rate of adverse reactions), and the *Q*-test and *I*^2^-test showed little heterogeneity (*P* = .93, *I*^2^ = 0%), using a fixed-effects model for analysis. The results showed that the rate of adverse reactions of acupoint application and sulfasalazine were comparable (RR 0.55, 95% CI 0.25–1.21, *P* = .14). TSA demonstrated that the current amount of information observed rate of adverse reaction was not conclusive and needed to be verified by more studies (Fig. 4).

### 3.5. Subgroup analysis

**Figure 5.**
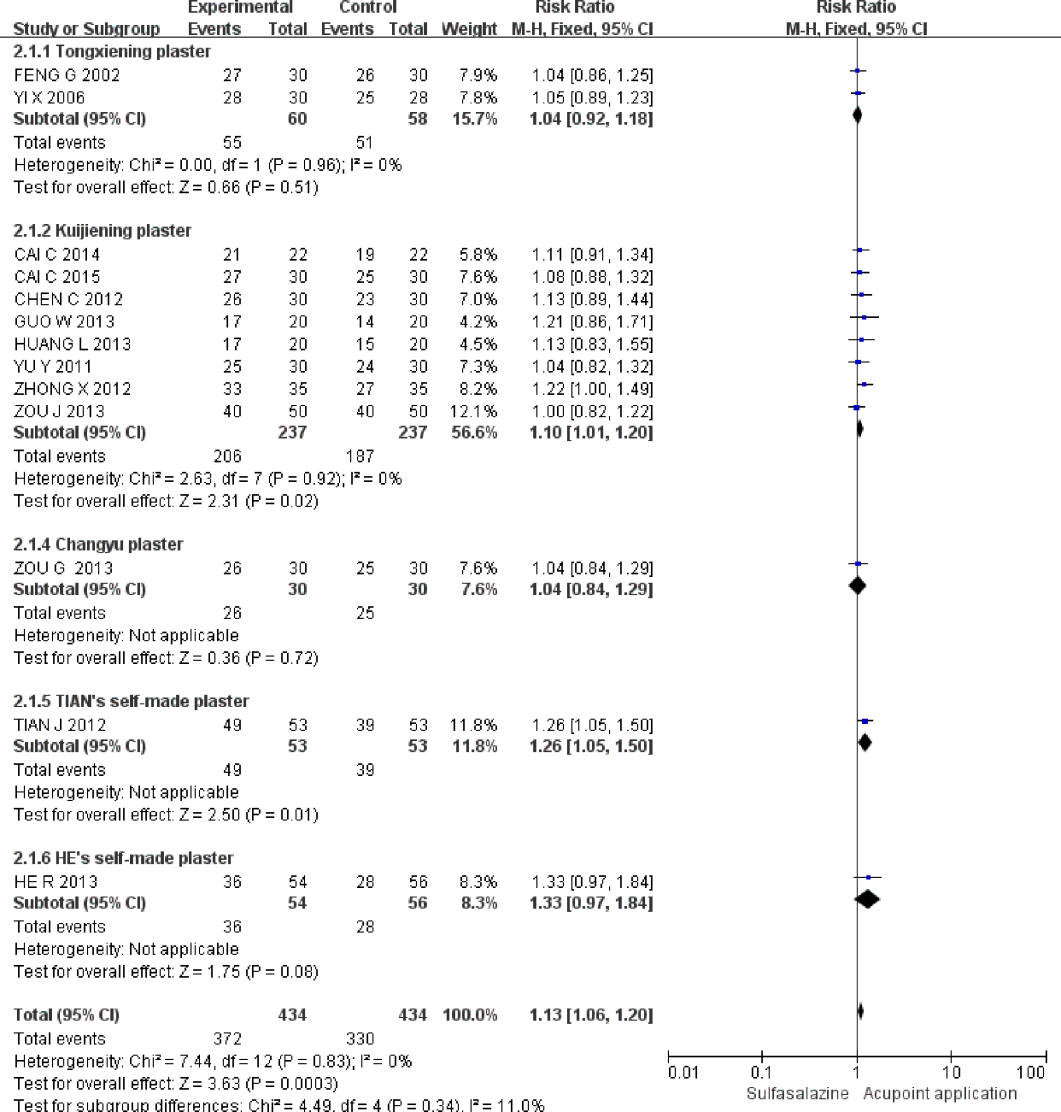
TSA of effective rate of clinical comprehensive of acupoint application in the treatment of UC. 95% CI = 95% confidence interval, TSA = trial sequential analysis, UC = ulcerative colitis.

Taking the composition of acupoint application prescriptions as the classification basis, five subgroups were constructed, namely “Tongxiening plaster,^[12,13]^” “Kuijiening plaster,^[14,15,17,18,20,22–24]^” “Changyu plaster,^[21]^” “TIAN’s self-made plaster,^[16]^” “HE’s self-made plaster.^[19]^” The effective rate of clinical comprehensive was used as the index to evaluate the effect of acupoint application with different prescriptions in the treatment of UC. The results showed that, compared with sulfasalazine, Kuijiening plaster (RR 1.10, 95% CI 1.01–1.20, *P* = .02), TIAN’s self-made plaster (RR 1.26, 95% CI 1.05–1.50, *P* = .01) can improve the effective rate of clinical comprehensive in the treatment of UC. However, Tongxiening plaster (RR 1.04, 95% CI 0.92–1.18, *P* = .51), Changyu plaster (RR 1.04, 95% CI 0.84–1.29, *P* = .72), and HE’s Self-made plaster (RR 1.33, 95% CI 0.97–1.84, *P* = .08) have comparable effective rate of clinical comprehensive (Fig. 5).

### 3.6. Publication bias assessment

**Figure 6.**
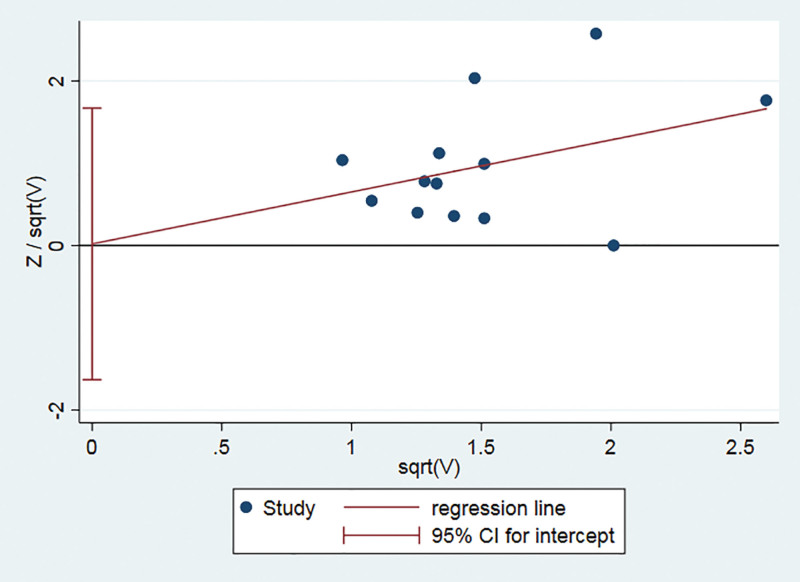
Harbord regression of the clinical comprehensive effective rate of acupoint application in the treatment of UC. 95% CI = 95% confidence interval, UC = ulcerative colitis.

Using the effective rate of clinical comprehensive as the standard, Harbord linear regression was performed to assess publication bias based on the event rates of the experimental group and the control group. The results showed no publication bias (*P* = .98) (Fig. 6).

### 3.7. Evidence quality assessment

Based on GRADE guidelines, GRADEpro3.6 software was used to evaluate the quality of evidence for the outcome indicators. The results showed that the quality of evidence of effective rates of clinical comprehensive and syndrome was moderate. The evidence quality of effective rates of colonoscopy and pathological examination, IL-4 and rate of adverse reaction was low, and the evidence quality of IFN-γ was very low. The recommendation strength was a weak recommendation (Table 3).

**Table 3:** Evidence quality assessment.

Outcomes	Limitation	Inconsistency	Indirectness	Imprecision	Publication bias	RR/MD(95% CI)	Quality of the evidence (GRADE)
Effective rate of clinical comprehensive	Serious*	No serious	No serious	No serious	None	1.13 (1.06–1.20)	Moderate
Effective rate of syndrome	Serious*	No serious	No serious	No serious	None	1.13 (1.03–1.24)	Moderate
Effective rate of colonoscopy	Serious*	Serious†	No serious	No serious	None	0.94 (0.84–1.05)	Low
Effective rate of pathological examination	Serious*	No serious	No serious	Serious‡	None	1.04 (0.90,1.20)	Low
IL-4	Serious*	No serious	No serious	Serious§	None	2.62 (1.96–3.28)	Low
IFN-γ	Serious*	Serious†	No serious	Serious§	None	−5.38 (−6.81 to −3.94)	Very low
rate of adverse reaction	Serious*	No serious	No serious	Serious‡	None	0.55 (0.25–1.21)	Low

IFN-γ = interferon-γ, IL-4 = interleukin-4, MD = mean differences, RR = risk ratio.

* Selection bias and implementation bias.

† Mild heterogeneity.

‡ Wide confidence interval.

§ Insufficient sample size.

## 4. Discussion

This study is the first systematic review of acupoint application for the treatment of UC so far, aiming to evaluate the clinical efficacy and safety of acupoint application compared with conventional western medicines such as sulfasalazine in the treatment of UC. In terms of clinical efficacy, meta-analysis reflected that acupoint application in the treatment of UC increased the effective rate of clinical comprehensive by 13% and syndrome by 13% compared with sulfasalazine. And the effective rates of colonoscopy and pathological examination are comparable to those of sulfasalazine. This means that the advantages of acupoint application over sulfasalazine may be reflected in the improvement of clinical syndromes. Meta-analysis also revealed that acupoint application increased the IL-4 by 2.62 ng/L and decreased the IFN-γ by 5.38 ng/L compared with sulfasalazine. That suggests that acupoint application was superior to sulfasalazine in reducing inflammation, which may improve the effective rates of clinical comprehensive and syndrome of treating UC by promoting the release of anti-inflammatory factors such as IL-4 and inhibiting the release of pro-inflammatory factors such as IFN-γ. TSA showed that the current results of the effective rate of clinical comprehensive were conclusive, and further clarified that the effective rate of clinical acupoint application in the treatment of UC was better than that of sulfasalazine. Harbord regression reflected that there was no obvious publication bias, and the quality of evidence evaluation showed that the quality of evidence for the effective rates of clinical comprehensive and syndrome was moderate, suggesting that the results of acupoint application benefit were more credible. Although the results of the meta-analysis negated the benefits of acupoint application on the effective rates of colonoscopy and pathological examination, the quality of evidence for the effective rate of colonoscopy was limited by the impact of inconsistency and the quality of evidence for the effective rate of pathological examination was limited by the impact of precision. The negative results of both may be due to insufficient sample size, so the results need to be tested by more high-quality studies.

The available studies suggest that the inflammation and tissue damage mediated by abnormal immune response is an important pathogenesis of UC,^[25]^ and autoimmune antibodies and inflammatory factors play a key role in this process.^[26]^ It has been shown in relevant studies that the mechanism of acupoint application in the treatment of UC mainly includes 2 aspects: Regulation of inflammatory factor levels: Acupoint application can reduce the levels of pro-inflammatory factors such as TNF-α, IL-4, IL-6, and IL-8, and increase the levels of anti-inflammatory factors such as IL-10, thus inhibiting the inflammatory response and reducing the inflammatory damage of the colonic mucosa in UC,^[14,27,28]^ as a way to improve the clinical symptoms and endoscopic manifestations of patients with UC. Modulation of autoimmune response: Acupoint application can effectively reduce serum IgA and IgG in patients with UC, and attenuate autoimmune damage to the colonic mucosa,^[29]^ thus improving the prognosis of patients with UC. The mechanisms of acupoint application for UC are poorly reported in the existing literature and need to be further explored in the future.

In terms of safety, meta-analysis shows that the rate of adverse reaction of acupoint application in the treatment of UC is comparable to that of sulfasalazine. In fact, the adverse reactions of acupoint application included in the studies are all skin damage caused by patients extending the application time without authorization. After excluding the cases of adverse reactions caused by irrational drug use, the meta-analysis showed that the rate of adverse reaction of acupoint application was significantly lower than that of sulfasalazine (RR 0.18, 95% CI 0.06–0.54, *P* = .002). It means that the safety of acupoint application in the treatment of UC may be better than that of sulfasalazine when the patients follow the prescribed medication.

Drugs and acupoints are the two elements for the clinical application of acupoint application. Table 4 summarizes the drug formulations and acupoint combinations included in the studies. In terms of drug selection, subgroup analysis showed that Kuijiening plaster and TIAN’s self-made plaster increased the effective rate of clinical comprehensive by 10% and 26%, respectively, while Tongxiening plaster, Changyu plaster and HE’s self-made plaster were comparable in effective rate of clinical comprehensive to sulfasalazine. It is suggested that Kuijiening plaster and TIAN’s self-made plaster may be relatively suitable drug formulations for the treatment of UC. But, the clinical effective rate of Tongxiening plaster, Changyu plaster and HE’s self-made plaster cannot be denied. Because FENG G et al and YI X et al also reported the advantages of Tongxiening paste in improving clinical symptoms; ZOU G et al pointed out the advantages of Changyu plaster in improving the cure rate; HE R et al also found the advantages of HE’s self-made plaster in reducing the recurrence rate, the clinical effective rate of Tongxiening plaster, Changyu plaster and HE’s self-made plaster needs to be further demonstrated by new research. Although the subgroup analysis confirmed the benefits of Kuijiening plaster and TIAN’s self-made plaster, only 1 report reported the effective rate of clinical of TIAN’s self-made plaster and no safety was reported. Yet, 8 studies reported the clinical efficacy of Kuijiening plaster and 6 studies reported its safety, so Kuijiening plaster may be the best drug choice for acupoint application based on the research status. The difference between these treatment schemes lies in the drugs and acupoints, and the specific ones are recorded in Table 4.

**Table 4:** Specific scheme of acupoint application for ulcerative colitis.

Scheme	Medicine	Acupoint
Tongxiening plaster	Coptidis rhizoma, Astragali radix, Atractylodis macrocephalae rhizoma, Vladimiriae radix, Angelicae sinensis radix, Paeoniae radix rubra, Rhei radix et rhizoma, Cinnamomi cortex	Zusanli, Pishu, Tianshu, Dachangshu
Kuijiening plaster	Aconiti lateralis radix Praeparata, Asari radix et rhizoma, Syzygium aromaticum, Semen sinapis, Corydalis rhizoma, Paeoniae radix rubra, Zingiber officinale roscoe, etc.	Shangjuxu, Tianshu, Zusanli, Minmen, Guanyuan
Changyu plaster	Coptidis rhizoma, Astragali radix, Rhei Radix et rhizoma, Paeoniae radix rubra, Cinnamomi cortex	Zusanli, Shenque, Zhongwan, Tianshu, Dangchangshu
TIAN’s self-made plaster	Semen plantaginis, Zanthoxylum bungeanum maxim, Cinnamomi cortex, Syzygium aromaticum	Shenque
HE’s self-made plaster	Asari radix et rhizoma, Syzygium aromaticum, Cinnamomi cortex, Folium artemisiae argyi, Dictamni cortex	Fengfu, Pishu,Shenshu, Shenque, Zusanli, Dachangshu

In terms of acupoint selection, all the acupoint combinations of Kuijiening plaster related research are “Shangjuxu, Tianshu, Zusanli, Mingmen, Guanyuan,” which are intended to warm and tonify the spleen and kidney. Two recent big data analysis^[30,31]^ showed that Tianshu, Zusanli, Shangjuxu, Guanyuan, and Zhongwan are the top five core acupuncture points for the treatment of UC with acupuncture. This is similar to the acupoint selection combination of Kuijiening plaster, which proves that the acupoint selection combination of Kuijiening plaster has good scientificity and reference value. In addition, data mining also found that Zhongwan is one of the core acupoints for the treatment of UC. In the future research of Kuijiening plaster, Zhongwan can be added to the acupoint combination scheme, which is expected to further improve its clinical efficacy. It is worth noting that the 8 included studies of Kuijiening plaster all limited the study subjects to patients with UC and spleen-kidney yang deficiency pattern, which means that the beneficiaries of acupoint application of Kuijiening plaster may mainly be patients with spleen-kidney yang deficiency pattern. To sum up, we recommend the acupoint application of Kuijiening plaster to warm and tonify the spleen and kidney as a routine method for the treatment of UC, which is supposed to become a new strategy for the treatment of UC.

The quality of evidence evaluation showed that the quality of evidence for 2 outcomes was moderate, the quality of evidence for 4 outcomes was low, and the quality of evidence for 1 outcome was very low, suggesting that this study has certain limitations. Firstly, there is a certain selection bias and implementation bias. Of the included studies, 3 studies had unclear risk of randomization methods, 12 studies had unclear risk of allocation concealment, and 10 studies had unclear risk of intervention blinding, all of which may have contributed to the formation of bias. Secondly, the limited sample size reduces the confidence of the results. The total number of samples for both IL-4 and IFN-γ was 240, and the small sample size reduced the reliability of the analysis results to a certain extent. Thirdly, narrow inclusion criteria reduced the generalizability of the results. YI X et al limited the included subjects to UC patients with internal damp-heat syndrome, and FENG G et al’s study was limited to UC patients with pattern of spleen deficiency and dampness-heat or with syndrome of intermingling of deficiency and excess, while 8 studies only included UC patients with spleen-kidney yang deficiency pattern. It means that the results of the meta-analysis mainly reflect the efficacy of acupoint application in the treatment of UC with spleen-kidney yang deficiency pattern, which limits the generalizability of the results. Fourthly, there are differences in the outcome measures of the studies. TIAN J et al and HE RM et al did not report the effective rate of syndrome of self-made plaster, so the syndromic efficiency cannot be used to explain the benefits of TIAN’s self-made plaster and HE’s self-made plaster. In the same way, the results of the effective rates of colonoscopy and pathological examination can only prove that the efficacy of colonoscopy and pathological examination of Kuijiening plaster is comparable to that of sulfasalazine, and it is not suitable for other acupoint application programs. Fifthly, among all the included studies, only “YI X 2006” clearly mentioned the inclusion of critically ill patients, and none of the other studies included critically ill patients or mentioned severity of illness. Therefore, this study alone cannot explain the benefit of acupoint application in critically ill patients. Sixthly, the follow-up period was relatively short. In the included studies, except HE RM et al, whose course of treatment was 12 months, the course of treatment for the rest of the studies was 1-2 months, suggesting that the results of the study mainly reflected the short-term efficacy of acupoint application in the treatment of UC. In fact, the clinical treatment of UC is a long-term medication process, and clinicians and patients pay more attention to the long-term effects of the treatment plan.

## 5. Conclusion

Acupoint application is a safe and effective method for the treatment of UC, which has the value of further research and exploration. However, due to the limitation of research base and research quality, the above results need to be verified by more high-quality clinical studies.

## Acknowledgments

We would like to thank Gaowen Wei for providing statistical guidance.

## Author contributions

**Data curation:** Shuang Yin.

**Methodology:** Yunfeng Yu.

**Software:** Shanzhi Lin.

**Supervision:** Yun Chen.

**Writing – original draft:** Yaling Tong.

**Writing – review & editing:** Xuan Su.
